# A Riemannian Modification of Artifact Subspace Reconstruction for EEG Artifact Handling

**DOI:** 10.3389/fnhum.2019.00141

**Published:** 2019-04-26

**Authors:** Sarah Blum, Nadine S. J. Jacobsen, Martin G. Bleichner, Stefan Debener

**Affiliations:** Neuropsychology Lab, Department of Psychology, European Medical School, Carl von Ossietzky University of Oldenburg, Oldenburg, Germany

**Keywords:** Riemann, mobile EEG, ASR, BCI, neuroergonomics, PCA, PGA

## Abstract

Artifact Subspace Reconstruction (ASR) is an adaptive method for the online or offline correction of artifacts comprising multichannel electroencephalography (EEG) recordings. It repeatedly computes a principal component analysis (PCA) on covariance matrices to detect artifacts based on their statistical properties in the component subspace. We adapted the existing ASR implementation by using Riemannian geometry for covariance matrix processing. EEG data that were recorded on smartphone in both outdoors and indoors conditions were used for evaluation (*N* = 27). A direct comparison between the original ASR and Riemannian ASR (rASR) was conducted for three performance measures: reduction of eye-blinks (sensitivity), improvement of visual-evoked potentials (VEPs) (specificity), and computation time (efficiency). Compared to ASR, our rASR algorithm performed favorably on all three measures. We conclude that rASR is suitable for the offline and online correction of multichannel EEG data acquired in laboratory and in field conditions.

## Introduction

Brain-Computer Interfaces (BCIs) translate brain signals into control signals and this translation is typically based on detecting different brain states in near real-time (Zander and Kothe, 2011; Yger et al., 2015). Non-invasive BCIs often rely on electroencephalography (EEG) for brain signal acquisition. EEG has the advantage that it can be recorded with small, unobtrusive and wireless hardware, incorporating off-the-shelf smartphones. Accordingly, portable EEG technology can bring BCI applications closer to real-life applications (Gwin et al., 2010; De Vos et al., 2014; Gramann et al., 2014; Debener et al., 2015; Jungnickel and Gramann, 2016; Bleichner and Debener, 2017; Blum et al., 2017). Moreover, EEG can be acquired while walking freely outdoors (Piñeyro Salvidegoitia et al., 2019) or indoors on treadmills (Solis-Escalante et al., 2012; Wagner et al., 2012; Seeber et al., 2015). However, while such recordings allow for the investigation of human behavior during motion and in natural environments, the resulting EEG signals are contaminated with even more artifacts than traditionally recorded, stationary EEG. In our previous studies, for instance, we observed that eye movements are more prominent in outdoors mobile compared to indoors stationary conditions. In the present paper, we considered stationary and mobile EEG data, recorded indoors and outdoors. Specifically, we evaluated two versions of an EEG artifact attenuation approach which can be applied online, that is, it can clean-up EEG artifacts in near real-time, a necessity for advancing BCI applications.

A widely used approach for artifact attenuation is independent component analysis (ICA) (e.g., Makeig et al., 1996; Delorme et al., 2007). Some studies show that ICA can be used online (Akhtar et al., 2012; Hsu et al., 2014; Pion-Tonachini et al., 2018), but it is computationally demanding and designed for offline use. ICA decomposes the EEG data into a set of maximally temporally independent components each representing different brain and non-brain sources. Components representing artifacts can either be removed manually or automatically (Campos Viola et al., 2009; Bigdely-Shamlo et al., 2013; Pion-Tonachini et al., 2018). ICA may not deal with all types of artifacts equally well, this holds in particular for non-biological artifacts that are difficult to model, such as cap movement or cable movement (Campos Viola et al., 2009; Chang et al., 2018). However, these artifact sources may compromise mobile EEG signal quality in particular.

Mullen et al. (2015) proposed an EEG artifact attenuation algorithm aimed at correcting anomalies in artifact-contaminated EEG data online. The ASR approach learns a statistical model on clean calibration data and attenuates (in theory any type of) artifact by decomposing short segments of EEG data and contrasting them to calibration data in the component subspace. ASR, like other statistical anomaly detection methods (Chandola et al., 2009; Islam et al., 2016), assumes that non-brain signals induce a large amount of variance in the EEG data and can therefore be detected based on their deviant statistical properties in the principal component analysis (PCA) subspace. Similar to ICA, data containing artifacts are reconstructed using an unmixing matrix to recover EEG data. ASR has been designed for online and offline use. In both cases, the data are processed in chunks of 500 ms. As a result, ASR has a very short processing delay and relatively low computational complexity. It is well-suited for online applications and it has been shown to perform very well on EEG data in different recording conditions (Arad et al., 2018; Kim and Kim, 2018; Plechawska-Wojcik et al., 2019).

In its original implementation, ASR decomposes covariance matrices of the EEG data for the detection of artifacts using traditional Euclidean geometry. Due to their mathematical properties though, covariance matrices should be processed using methods applicable for curved, high-dimensional data spaces (Moakher and Batchelor, 2006; Yger et al., 2015; Lotte et al., 2018). For such computations, Riemannian geometry has been shown to be more precise than Euclidean methods (Barachant et al., 2012, 2013; Kalunga et al., 2015; Yger et al., 2015; Horev et al., 2017; Lotte et al., 2018) and has been used in online artifact detection and correction previously (Barthélemy et al., 2017, 2019). Therefore, we speculated that a Riemannian-adapted ASR could outperform the original ASR algorithm. In this report, we describe the implementation and evaluation of a Riemannian version of ASR, hereafter referred to as rASR. Specifically, we compared rASR to ASR and to uncorrected EEG data. As measures of artifact reduction sensitivity, we evaluated the performance by which ASR and rASR reduce eye-blink amplitudes and topographies. As measures of artifact reduction specificity, we calculated and compared visual-evoked potential (VEP) signal-to-noise ratios (SNR) and amplitudes. Moreover, computation times for each procedure were measured to assess computational efficiency.

We used 24-channel EEG data recorded from *N* = 27 participants performing a memory experiment similar to the one described in Piñeyro Salvidegoitia et al. (2019). EEG acquisition and experimental control were implemented on a smartphone, and artifact correction performance was evaluated for two conditions, standing and walking outdoors on the university campus, and standing and walking indoors on a treadmill.

## Materials and Methods

### The Original ASR Algorithm and the EEGLAB Plugin *clean_rawdata*

The ASR algorithm is explained in detail in Chang et al. (2018), Mullen et al. (2015), and Pion-Tonachini et al. (2018) and is available as part of the open source EEGLAB plugin *clean_rawdata*^1^. Briefly, ASR learns statistical properties of clean calibration data and compares these statistics during the processing with statistics of new data, potentially containing artifacts. It is recommended that the calibration data have a length of at least 1 min and be recorded from the respective participant during rest under comparable recording conditions as the subsequent processing. The ASR algorithm consists of two parts, the calibration and the processing. During the calibration, data are filtered, then a robust covariance matrix U, ∈ ℝ^c×c^, with c being the number of channels, is computed using the L1-median of subsequent sample covariance matrices of the incoming data segment. The geometric median, or L1-median is defined as U = ${\mathrm{argmin}}_{U}$$\sum_{i=1}^{n}{||X_{i}\,X_{i}^{T}-U||}_{2}$, where *argmin* minimizes the Euclidean distance of X to U and n denotes the amount of covariance matrices computed so far during the calibration. The single sample covariance matrices are computed as Cov (s) = X (s) X (s)^T^, Cov (s) ∈ ℝ^c×c^, with c being the channel number, X (s) ∈ ℝ^t×c^ the data segment, s consisting of c channels and t samples. U is then used to compute the mixing matrix M ∈ ℝ^c×c^ such that MM^T^ = U. While learning the statistical model, this mixing matrix M is decomposed using PCA to obtain V ∈ ℝ^1×c^ and D ∈ ℝ^1×c^, the eigenvectors and eigenvalues of the mixing matrix. At the end of the calibration phase, data is projected into component space where statistical properties of each component are computed. These statistics are used to determine a threshold operator T = μ + k × σ, T ∈ ℝ^c×c^, with μ, σ being mean and standard deviation of the components and k a tuning parameter, evaluated in Chang et al. (2018). The threshold matrix T defines the limits of normal data during the processing. During the processing, the current covariance matrix Cov (s) is averaged with the recording from the previous data segment s that was being processed. Those matrices are temporally smoothed, U(s) = f(cov(s), U(s – 1)) where U (s) ∈ ℝ^c×c^ depends on the chosen step size parameter for the data segment *s* and *f* is a weighted Euclidean average function. The amount of covariance matrices in U that are decomposed in succession depends on the window length parameter and the amount of samples currently in the method: range = sampling rate ^∗^ windowlength (500 ms by default). range defines the size of U : C × C × range, the update interval used for decomposition is defined by $\frac{\mathrm{range}}{\mathrm{stepsize}}$ which translates to several thousand iterations of decomposition and reconstruction for every data segment in the ASR method. During this main processing loop, U is indexed in chunks of size stepsize, those U(s) ∈ ℝ^c×c^ are used to detect and reconstruct artifacts. In component space, data are reconstructed using the mixing matrix M from the calibration and a portion of the eigenvectors V _clean_ of U(s), namely those of the clean components determined by the threshold operator T: $X_{\mathrm{clean}}=M\,{\left(V_{\mathrm{clean}}^{T}\,M\right)}^{+}\,V^{T}\,X$. As a result, the artifact is removed from the data. For a complete description of implementation details, please refer to the *asr_process* function in the *clean_rawdata* plugin.

### Overview of Riemannian Geometry for EEG Data

The central step in both ASR and rASR is the computation and decomposition of covariance matrices using an eigendecomposition. Data is thereby represented on a new set of axes which fulfill a statistical criterion (Clifford, 2008). In the case of eigendecomposition of covariance matrices, this criterion is variance. Covariance matrices are symmetric positive definite (SPD) matrices which lie in the space M(n) of all n x n real matrices (Lang, 1999), which is a differentiable Riemannian manifold (e.g., Barachant et al., 2012). In this space, they belong to a subset which forms a convex cone (Dattorro, 2017). Several studies have shown the beneficial effects of using Riemannian distance and average measures, as well as geometry-aware PCA methods, such as principal geodesic analysis (PGA) (Fletcher et al., 2004; Horev et al., 2017). Since the space of SPD matrices is a (negatively) *curved space*, the use of traditional Euclidean geometry, which implies that distances are computed along straight lines in the data space, turns out to be disadvantageous. Riemannian geometry has shown to be more efficient and yields more precise results in the analysis of EEG data (Shyu et al., 2006; Kalunga et al., 2015; Yger et al., 2015; Congedo et al., 2017; Horev et al., 2017; Barthélemy et al., 2019).

### The rASR Algorithm: Geometry Aware Methods

The rASR algorithm differs from original ASR in all computations or decomposition of covariance matrices during the processing. In rASR, a sample covariance matrix is computed as a robust, unbiased estimator of the covariance matrix of the current data segment (Kalunga et al., 2015). It is defined as: $U=\frac{1}{t-1}\,{\mathrm{XX}}^{T},$ where t is the number of samples in the current data segment and X ∈ ℝ^t×c^ is the current channel matrix consisting of t samples and c channels (Barachant et al., 2012; Barthélemy et al., 2019). By computing an estimator of the covariance matrix of the current data segment held in the method, the rASR method omits the necessity of computing individual covariance matrices for every small chunk of the data defined by stepsize, as described in the ASR paragraph. As a result, instead of decomposing several thousand covariance matrices, this expensive operation is only done once on the estimator covariance matrix. The reconstruction of the data segment is then done successively in small chunks, the same as in the original ASR method. Here, the stepsize parameter is set to a smaller value in rASR to ensure a good coverage of reconstruction and counterbalance the less-sensitive covariance matrix computation. To ensure a robust covariance matrix for successive data segments, the currently computed covariance matrix is, both in rASR as well as in ASR, averaged with the covariance matrix computed with the previous data segment. The running mean averaging method used in ASR potentially induces imprecisions in the result because of the so-called *swelling effect*, which can occur when using Euclidean geometry in non-linear spaces (Horev et al., 2017). In rASR, the method of averaging the current and previous covariance matrices was therefore exchanged by a geometry-aware averaging method, namely the Riemannian center of mass method, also known as Karcher mean (Horev et al., 2017): $U\,\left(s\right)={\mathrm{argmin}}_{U}\,\sum_{i=0}^{N}d\,{\left(\mathrm{Cov}\,\left(s-i\right),U\right)}^{2}$, where d is the geodesic (Riemannian) distance which is minimized, the analogous concept to Euclidean distance in the arithmetic mean. In rASR, the computation of the sample covariance matrices is therefore done differently and in addition, it is done less often because the sample covariance estimator is used instead of covariance matrices of many samples of the current data segment. Further, the eigendecomposition is computed using a PGA instead of a PCA, described in Fletcher et al. (2004) and Horev et al. (2017) and implemented in the Manopt toolbox (Boumal et al., 2013). PGA extends PCAs dimensionality reduction to SPD matrices. The goal in PGA is to project data into a lower dimensional subspace which best preserves the variance, analogous to PCA. In PGA, the data are projected to geodesic submanifolds which are computed such that they maximize the projected variance of the data. Distances between submanifolds are given by geodesics, the analogous of a straight line in curved space. Variance is defined as the Riemannian distance from the mean, linear subspaces from PCA are now extended to geodesic manifolds (an extensive explanation of PGA can be found in Fletcher et al. (2004)). These distances between components are especially large when data contain artifacts and component matrices differ considerably from each other, as assumed in the case of an artifactual data segment, leading to large distances on the manifold. In this case, PGA preserves more variance than PCA (Horev et al., 2017).

### Experimental Design and Procedure

An unpublished data set recorded for a different purpose was used in the present report (Jacobsen, unpublished). This study followed a previous work of our group investigating the neural correlates of episodic memory formation outdoors (Piñeyro Salvidegoitia et al., 2019). The study was approved by the local ethics committee, participants provided their written informed consent prior to participation. We recorded EEG data from 27 subjects (7 male, 20 female, mean age 23 years, ± 2.5 years SD) recorded in two conditions, one indoors and one outdoors. A smartphone (“Sony Xperia Z1,” model: C6903; OS: Android 5.1.1) running the recording application Smarting (version 1.6.0, mBrainTrain, 2016 Fully Mobile EEG Devices) and a 24-channel mobile EEG cap (Easycap, Herrsching, Germany) were used to collect and record EEG data. The Presentation mobile app (Version 1.2.1, Neurobehavioral Systems Inc., Albany, CA, United States, **RRID:SCR_002521**) controlled experimental events. In the outdoors condition, participants were instructed to walk naturally next to the experimenter who guided them to different locations on the university campus. Indoors, participants walked on a mechanic treadmill at their preferred speed. In both conditions, words that subjects had to remember were presented on the smartphone display. The experimental design involved self-initiated presentation of those words, subjects first stopped walking and then pressed a button on the screen to start a trial. Outdoors, participants stopped walking when indicated by the experimenter, indoors, participants stopped walking when indicated by a cue on the smartphone display. Note that the data used for the analyses of the VEP elicited by the word on the screen were therefore recorded when subjects stood still and looked at the smartphone screen. Eye-blink data on the other hand were extracted from the entire indoor and outdoor dataset, containing data recorded while participants were walking and while they were standing. In the beginning of each block, 1 min of resting EEG was recorded which served as calibration data for ASR and rASR. Matlab [Version 9.0 (2016a), The Mathworks Inc., Natick, MA, United States, **RRID:SCR_001622**] and EEGLAB (Version 13.6.5b, Delorme and Scott, 2004, **RRID:SCR_007292**) were used for all analyses. The rASR toolbox was developed by the first author of this report (SB) and is freely available alongside all processing scripts used for our analyses^2^.

### Preprocessing

**Figure 1:**
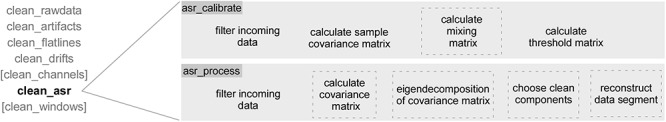
The EEGLAB toolbox methods are listed on the left (in gray) and include the two core functions of ASR and rASR that are called by the function *clean_asr*. Parts of the algorithm that were adapted using geometry-aware methods are marked by dashed sections. The toolbox consists of several preprocessing and correction functions that together comprise artifact correction for multi-channel EEG data. Some of the functions marked in square brackets are computationally expensive and profit from many data, but they are typically not used for online applications, we therefore refrained from using them herein. When using the toolbox, all functions are called once, *clean_asr* is then calling the calibration and subsequently the processing repeatedly with short segments of the uncorrected data. The procedure is the same for the online and offline use of ASR and rASR.

All data were recorded with a sampling rate of 250 Hz, digitized data recordings had a resolution of 24 bit. Passive Ag/AgCl electrodes were used and arranged according to the international 10/20 setup (reference: FCz, DRL: AFz). Impedances were kept below 10 kΩ. EEG data, experimental events and additional sensor readings from the smartphone and IMUs in the amplifier were recorded into an *xdf* file on the smartphone^3^. For subsequent analyses, the EEG data were low-pass filtered at 40 Hz (FIR, filter order 166) and high-pass filtered at 0.25 Hz (FIR, filter order 3300). Data were then submitted to a subset of functions from the *clean_rawdata* toolbox which contains the ASR algorithm (version 0.34). rASR is implemented in its own plugin which is identical to *clean_rawdata* in all but the two core methods *asr_calibrate* and *asr_process*. Figure 1 shows the toolbox and its wrapper and preprocessing functions for the ASR algorithm. The ASR algorithm is implemented in the core functions *asr_calibrate* and *asr_process* which are therefore the only functions that were changed to develop a Riemannian-adapted ASR algorithm. In Figure 1, adapted parts in rASR indicated by dashed outlines. During the processing, segments of raw EEG data are corrected in several steps which have all been adapted in the rASR algorithm. The covariance matrix computation and decomposition now use geometry-aware methods, the choice of clean components and the reconstruction are called in different order but have not been methodologically adapted. This rearrangement and replacement of parts of the ASR algorithm requires different parameters in the call of *clean_asr* for ASR and rASR, whereby rASR needs substantially more aggressive values in particular because of the scarcer update of the covariance matrix. For both ASR and rASR, we cleaned the calibration data recorded at the beginning of the experimental blocks using ASR or rASR before submitting them to processing. Data from each block were then processed using *clean_flatlines*, *clean_drifts*, and finally *clean_asr* using the dedicated cleaned calibration data. Since we are aiming for an online artifact cleaning solution, we refrained from using *clean_channels*, a powerful, yet computationally expensive and time-consuming method which cannot be used in online processing of EEG data. ASR was first used with the pre-defined default parameters, but after cleaning of the calibration data before submitting it to the processing, these parameters yielded results of clear overcorrection, so they were set to less aggressive parameters. Parameters for both methods were evaluated before starting the analysis by means of maximizing SNR and preserving morphology and are specified hereafter with name (value for rASR, value for ASR): flatline (1, 5), hp [(0.25, 0.95), (0.25, 0.95)], channel (0.9, -1), noisy (3, -1), burst (2, -1), window (0.3, 0.5), cutoff (1, 5), stepsize (16, 32), maxdims (1, 0.66). All remaining parameters were used with their default values.

Apart from the artifact attenuation step, the analysis pipeline was identical for both methods. Data were epoched according to events in the data, either stemming from experimental events (VEP) or from detected artifact events (eye-blinks). For comparison reasons, we also included data which were temporally filtered the same way as the artifact-corrected data sets but otherwise not further processed. These data are further referred to as *uncorrected* data.

### Specificity Analysis: VEP

An artifact correction method that reduces the overall data amplitude will inevitably reduce artifact amplitudes, as well as the amplitudes of any signal of interest. In order to examine whether a signal of interest is retained in the corrected EEG data, we analyzed the amplitude and the SNR of VEPs. We compared ASR, rASR and uncorrected data for both indoors and outdoors data. We focused our analyses on VEPs elicited by words appearing on the smartphone screen. These onset/offset VEPs evoked by a static stimulus have a distinct morphology in time and space (Odom et al., 2016). We analyzed data recorded by occipital channels O1 and O2, referenced to the analog reference (located at FCz). Furthermore, every subject’s average VEP was used to determine individual indices for the latencies of the early positive deflection and the following negative deflection. The SNR was computed by dividing the ERP peak amplitude by a pre-baseline noise estimator of the standard deviation in the pre-stimulus interval (200 ms before stimulus trigger). SNR is reported in decibel (dB) throughout this paper: $\mathrm{dB}=10^{*}\,\log_{10}\left(\frac{\mathrm{signal}}{\mathrm{noise}}\right)$. To compare the specificity in retaining the signal of interest for the artifact attenuation procedures, VEP SNR values and amplitudes were statistically analyzed using a 2 × 3 repeated measures analysis of variance (ANOVA) with the factors condition (indoors, outdoors) and method (uncorrected, ASR, rASR) and followed up by paired *t*-tests where appropriate.

### Sensitivity Analysis: Eye-Blink Reduction

In order to objectively identify eye-blinks, we used the freely available Blinker toolbox (Kleifges et al., 2017) to detect eye-related artifacts on data from the combined frontal channels Fp1, Fp2. Time indices of detected eye-blinks were added to the data sets as eye-blink events and were then used to create eye-blink epochs. The average number of eye-blinks per subject was 1165 during the outdoors condition (range: 642 to 2013) and 962 indoors (range: 361 to 1813). Blink amplitudes were statistically analyzed using a 2 × 2 repeated measures ANOVA with factors condition (indoors, outdoors) and method (ASR, rASR) to assess whether one method outperformed the other, and whether this was the case for both indoors and outdoors data. Significant ANOVA effects were followed up by paired *t*-tests.

In addition to the eye-blink amplitude analysis, we also evaluated eye-blink topography residual variance. Assuming spatial orthogonality between ongoing EEG and eye-blinks, a complete reduction of eye-blinks should reduce the correlation of eye-blink topographies before and after the correction to a minimum, whereas an incomplete (or over complete) correction would be indicated by residual absolute correlation between topographies of uncorrected and corrected data. Accordingly, we calculated average eye-blinks for each condition and derived the topography at eye-blink peak latency in uncorrected and corrected data sets. Pearson correlation values were Fisher z-transformed and submitted to a 2 × 2 ANOVA with the factors condition (indoors, outdoors) and method (ASR, rASR).

## Results

### Specificity: Visual-Evoked Potential

**Figure 2:**
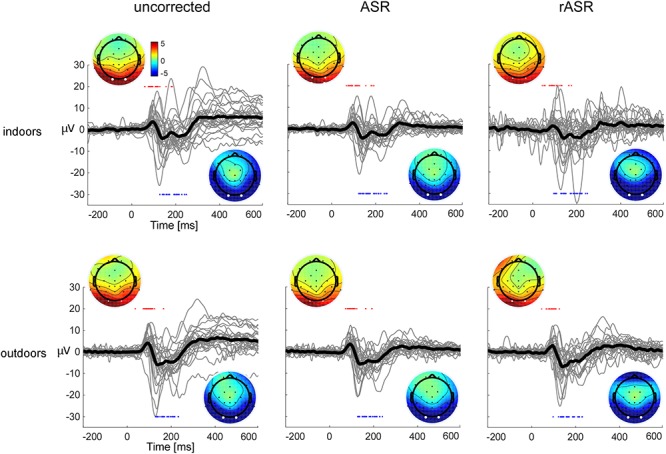
Single subject (gray lines) and grand average (black lines) visual-evoked potentials, shown for uncorrected data (left) and following correction with ASR and rASR (center column and right). VEPs are shown for indoors and outdoors data and show the arithmetic mean of occipital channels O1 and O2, referenced to a central reference near Cz. The voltage maps show the averaged single subject topographies at subject-specific latencies for the early P1 and the later N1 response, all topographies are scaled identically. Individual latencies are indicated by red (P1) and blue (N1) dots. In the uncorrected data, one channel (right temporal T8) was interpolated for the voltage map.

Figure 2 shows the averaged single subject VEPs together with the grand averaged VEP P1 and N1 topographies for both conditions, uncorrected data and ASR and rASR corrected data. We observed typical bilateral occipital VEP topographies in corrected and uncorrected data. As can be seen, ASR and rASR did not affect VEPs very much. The similarity of VEPs between indoor and outdoor conditions complements previous reports on outdoor ERP acquisitions (Debener et al., 2012; De Vos et al., 2014) and suggests that VEPs can be obtained from visual stimuli presented on smartphone, even in otherwise uncontrolled outdoor conditions. ASR and rASR preserved the morphology of the VEP and reduced the standard deviation of the signal. The VEP maps revealed the expected early positive and late negative occipital activation in both conditions, indoors and outdoors. The amplitudes of the VEP N1 peaks were lower in ASR corrected data sets than in uncorrected and rASR corrected data sets (uncorrected: -8.5 μV indoors, -10.35 μV outdoors, ASR: -8.1 μV indoors, -9.56 μV outdoors, rASR: -10.44 μV indoors, -10.61 μV outdoors). A 2 × 3 ANOVA of the N1 peak amplitude with the two within subjects factors, condition (indoors, outdoors) and method (uncorrected, ASR, rASR) yielded a significant main effect for condition [*F*(1,26) = 5.33, *p* = 0.03, ηp^2^ = 0.01]. VEP amplitudes were smaller indoors (-9.11 μV) than outdoors (-10.4 μV). We found no evidence for a difference between methods [*F*(2,52) = 1.49, n.s.]. A comparable 2 × 3 ANOVA of the N1 SNR with two within subjects factors, condition (indoors, outdoors), and method (uncorrected, ASR, rASR) yielded no significant results, neither for the main effect condition [*F*(1,26) = 1.19, n.s.], nor for the main effect method [*F*(2,52) = 1.25, n.s.]. The interaction effect missed significance as well [*F*(2,52) = 2.91, *p* = 0.06, n.s.] and was therefore not further examined.

### Sensitivity: Blink Amplitudes

**Figure 3:**
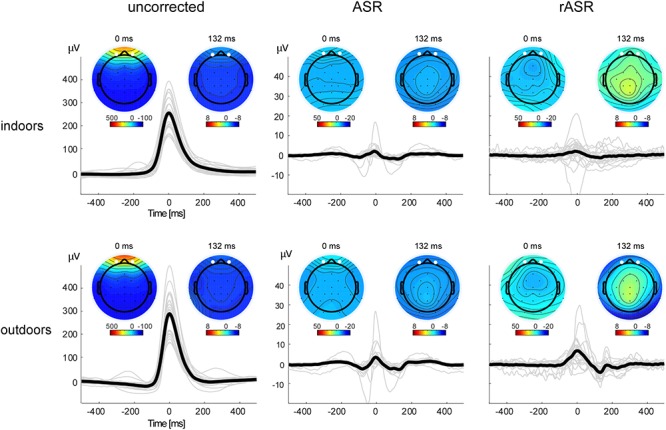
Blink artifact topographies and time courses for all methods and conditions. Note the different *y*-axis scale for uncorrected and corrected data. The bold black lines show the grand average blink signal, the thin gray lines show single subject averages. Eye-blink potentials were measured as the mean between channels Fp1 and Fp2, marked as white points in the voltage maps. Blink potentials and blink voltage maps illustrate eye-blink morphologies and topographies before (left) and after (center, right) artifact correction. Smaller maps show the voltage distribution 132 ms post eye-blink maximum. In uncorrected data, the map reflects residual eye-blink activity, in corrected datasets, it is compatible with the interpretation of eye-blink generated event-related brain potentials.

Figure 3 shows the ASR and rASR comparison to uncorrected data for the analysis of the blink amplitudes. As expected, the blink amplitude of uncorrected data were orders of magnitudes larger than typical EEG signals of interest with a mean of 254.92 μV indoors and 289.02 μV outdoors. A 2 × 2 repeated measures ANOVA with two within subject factors condition (indoors, outdoors) and method (ASR, rASR) yielded a significant main effect for condition. The residual blink amplitude was significantly smaller indoors (2.33 μV) than outdoors [5.37 μV , *F*(1,26) = 8.28, *p* = 0.008, η*p*^2^ = 0.07] but no significant difference between methods was found [*F*(1,26) = 2.56, *p* = 0.12].

**Figure 4:**
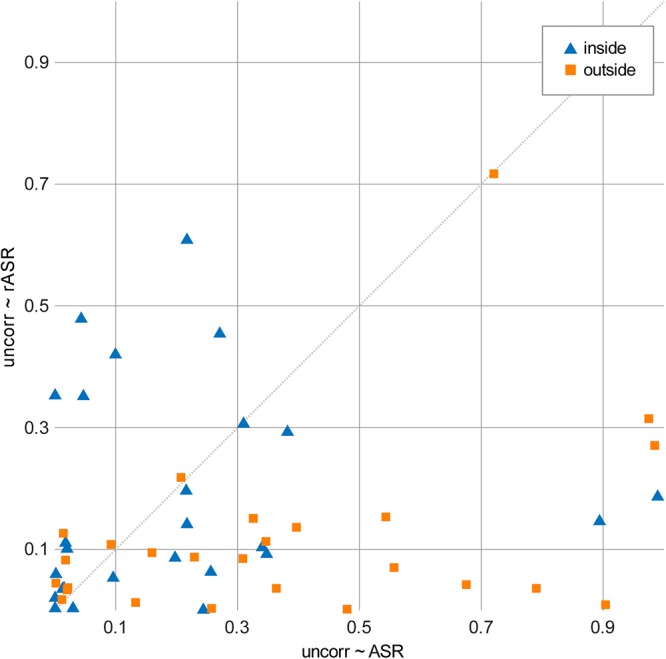
Similarity values (R^2^) of eye-blink topographies obtained by correlating uncorrected and corrected topographies. A low R^2^ value reflects very little residual topographical influence of blinks, thereby indicating better artifact suppression. Comparison of correlation values between uncorrected and ASR-corrected topographies (*x*-axis) and between uncorrected and rASR-corrected topographies (*y*-axis). The diagonal line marks the border between favorable values for rASR (lower section) and favorable values for ASR (upper section). rASR showed lower similarity values than ASR.

In addition to the blink amplitude on the frontopolar channels alone, we also evaluated blink topographies, similar to Campos Viola et al. (2009) and Bigdely-Shamlo et al. (2013), following the notion of a multidimensional description of artifacts (Tamburro et al., 2018). Figure 4 shows similarity values (R^2^), reflecting the shared variance between blink topographies of uncorrected and corrected data sets. This was calculated separately for indoors and outdoors conditions and both artifact correction methods. Statistical evaluation was performed with Fisher z correlation values of corrected and uncorrected topographies. The corresponding 2 × 2 ANOVA with two within subject factors (indoors, outdoors) and method (ASR, rASR) yielded a main effect for method [*F*(1,26) = 10.42, *p* = 0.003, ηp^2^ = 0.08]: the average similarity value was significantly higher for the ASR method (mean R^2^ = 0.283) than for the rASR method (meanR^2^ = 0.15). The main effect of condition was not significant [*F*(1,26) = n.s.], however, the interaction between method and condition was significant [*F*(1,26) = 0.83, *p* = 0.004, η*p*^2^ = 0.06], indicating a significant difference of methods outdoors [*t*(26) = -4.51, *p* = 0.00012, *d* = 1.03] but not indoors [*t*(26) = n.s.].

Following eye-blink correction we observed small post eye-blink deflections that could either indicate residual artifact or eye-blink related cortical potentials. As indicated by topographical inspection, a negative deflection approximately 132 ms post eye-blink peak latency was evident. Similar effects have been described before (Berg and Davies, 1988; Heuser-Link et al., 1992) as indicating a cortical response to the lid closure. Since this effect was unpredicted, we did not follow it up statistically.

### Computation Time

**Figure 5:**
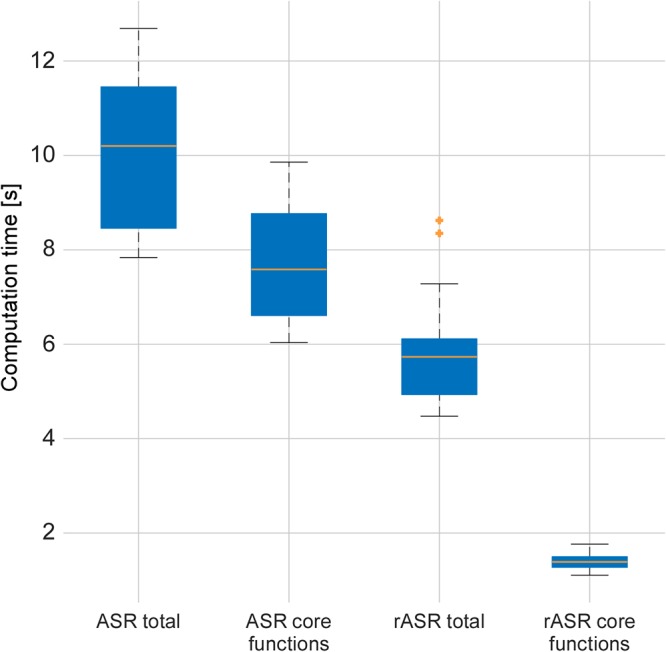
Artifact correction computation times per method and extend of used functions for the two artifact-correction procedures, measured on a laptop running Matlab 2016a, Intel^®^i7 (quadcore) processor and 16GB RAM. The median is shown by the line which divides the boxes, outliers are indicated by crosses outside the whiskers. The core functions contained the Riemannian adaptation and therefore show the biggest difference in computation time. When computation time for all preprocessing functions was measured, the difference was less pronounced since these functions include unchanged computations.

Matlab was used to measure the processing time to correct the data sets using the different methods, once including all toolbox calls and once regarding only the core functions (i.e., the ones that were adapted). As illustrated in Figure 5, when measuring the computation time including all wrapper and preprocessing functions contained in the toolbox, rASR took 44.5% less time to process 158 data sets (27 participants with 4 to 6 blocks of recording) than ASR (mean rASR = 5.6 s, *SD* = 0.7 s, mean ASR = 10 s, *SD* = 1.5 s per data set). When only measuring the time for the core algorithm, rASR was even more efficient. This is because this measurement only includes those functions that contained the Riemannian adaptation. Here, rASR took 82% less time to process the data sets than ASR (mean time rASR = 1.4 s, *SD* = 0.1 s, mean time ASR = 7.7 s, *SD* = 1.1 s per data set). A paired *t*-test confirmed significant differences between the methods, both for the complete computation time [*t*(157) = -55.15, *p* < 0.01, *d* = 4.39] and the minimal computation time [*t*(157) = -77.51, *p* < 0.01, *d* = 6.17].

## Discussion

Fast and powerful artifact correction methods are needed for EEG applications, especially for mobile recordings. This requires a computationally efficient procedure and an approach that can deal with the complexity of EEG recorded in mobile conditions, such as walking outdoors. We have extended the original ASR algorithm with Riemannian geometry. The use of Riemannian methods in the computations involving covariance matrices proved to be beneficial, rASR was faster than ASR and corrected blink artifacts favorably to ASR. At the same time, rASR preserved the signal of interest, here the visually-evoked potential VEP. However, the following issues require consideration.

Regarding artifacts, we focused on eye-blinks and disregarded other physiological or non-physiological artifacts. The reason for this choice was that eye-blinks have a strong impact on event-related EEG analysis quality as they have a large amplitude, dominating multi-channel recordings at time of occurrence. Moreover, eye-blinks have been well characterized physiologically and tools exist that can automatically detect eye-blinks with reasonably good accuracy. We used the Blinker toolbox (Kleifges et al., 2017) to identify eye-blinks and created eye-blink artifact related epochs, in order to evaluate eye-blink correction quality. By focusing on automatically detected, we may have missed non-stereotypical blinks that were not detected by the Blinker toolbox because a coherent blink template could not be learned for them. We did not evaluate how well ASR and rASR perform on these non-stereotypical eye-related artifacts. While future work may benefit from improved blink detection procedures (Barthélemy et al., 2017; Jas et al., 2017) and the inclusion of other EEG artifacts to provide a more complete picture, the current analyses suggest that rASR implements eye-artifact correction very successfully for common eye-blinks, regardless of whether they were recorded indoors or outdoors.

Interestingly, eye-blink correction did not result in isoelectric activity, even after averaging over a larger number of eye-blink epochs. This suggests that either the correction procedure applied under- or overcorrected the artifact, or that an event-related brain potential in response to eye-blinks was found. The topographical analysis as illustrated in Figure 3 supports the latter interpretation. Blink-related event-related potentials have been reported before (Berg and Davies, 1988; Heuser-Link et al., 1992) but did not receive much attention lately. However, eye-blinks provide information about cognitive processing (Wascher et al., 2015) and, in combination with a thorough analysis of blink-generated event-related potentials, may provide a rich source of information in applied fields, such as neuroergonomics.

It is worth noting that ASR and rASR benefit from the recording of suitable calibration data. One minute of resting EEG containing few artifacts is recommended (Mullen et al., 2015). In our experience the quality of the calibration data plays an important role in the quality of ASR-based artifact reduction, and this probably also holds for the modified version of rASR. However, it was beyond the scope of this paper to compare the role of the calibration data on ASR and rASR performance. We recommend using proper calibration data, that is, calibration data taken from the same recording session and based on resting conditions. ASR performance on attenuating particular artifacts, such as free walking-related artifacts may critically depend on the same artifact being absent in the calibration data. In a future project, it would be very interesting to specifically investigate the artifact reduction capabilities of rASR on movement-related artifacts in general and walking-related artifacts in particular (Gwin et al., 2010; Oliveira et al., 2016). The mobile EEG system used in the present study features the concurrent recording of 24-channel EEG, 3D gyroscope signals from the head and 3D accelerometer signals from the smartphone, and these motion sensor signals, in particular when located at the feet, may be instrumental for gait-artifact detection evaluation (Hwang et al., 2018). The current gold standard in EEG artifact reduction seems to be ICA, which was shown in a number of studies to perform favorably when compared to other artifact reduction procedures (Hoffmann and Falkenstein, 2008; McMenamin et al., 2010) and with suitable parameters (Winkler et al., 2015). However, only few attempts were made to apply ICA online (Akhtar et al., 2012; Hsu et al., 2014; Pion-Tonachini et al., 2018). These particular approaches are computationally expensive and require careful validation of block size and sensitivity measures. To our knowledge, previously presented online EEG artifact suppression procedures appear to be more complex and computationally more demanding than ASR and rASR. Accordingly, it is not clear whether these procedures could be operated on handheld devices such as smartphones. The rASR algorithm presented here is computationally less expensive than ASR and introduces only a small processing lag. We are therefore convinced that future mobile EEG applications will benefit from efficient online artifact correction. Of course, online artifact correction may not perform well under any circumstances, therefore it should be good practice to apply state-of-the-art artifact correction offline and compare the results. This requires software solutions to save both artifact-corrected as well as uncorrected raw data. To support a widespread use of online artifact correction, the original ASR artifact correction method was implemented in Java and will be included in SCALA (Blum et al., 2017), a fully mobile framework for BCI applications on smartphone. SCALA is freely available on GitHub for download and is considered an open beta. It uses the Lab Streaming Layer (Swartz Center for Computational Neuroscience and Kothe, 2015) to receive raw data and event markers. SCALA classifies incoming time series such as EEG online and returns a classification result via LSL. The rASR algorithm presented here will be included into SCALA in the near future.

## Author Contributions

SB implemented rASR and wrote the analysis scripts. NJ collected the data in a previous study in our group. SD supervised the project during which the data were originally collected. SD and MB supervised this project. All authors contributed to the analysis of the results and to the writing of the manuscript.

## Conflict of Interest Statement

The authors declare that the research was conducted in the absence of any commercial or financial relationships that could be construed as a potential conflict of interest. The handling Editor declared a past co-authorship with one of the authors SD.

## Footnotes

1 https://sccn.ucsd.edu/wiki/EEGLAB_Extensions

2 http://github.com/s4rify/rASRMatlab

3 https://github.com/sccn/xdf/wiki/Specifications
